# Introducing an Optimal Liver Allocation System for Liver Cirrhosis Patients

**DOI:** 10.5812/hepatmon.10479

**Published:** 2013-08-30

**Authors:** Jamileh Abolghasemi, Mohammad Reza Eshraghian, Mohsen Nasiri Toosi, Mahmood Mahmoodi, Abbas Rahimi Foroushani

**Affiliations:** 1 Department of Epidemiology and Biostatistics, School of Public Health, Tehran University of Medical Sciences, Tehran, IR Iran; 2 Department of Gastroenterology, School of Medicine, Tehran University of Medical Sciences, Tehran, IR Iran

**Keywords:** Liver Transplantation, End-Stage Liver Disease, Allocation, Liver Cirrhosis

## Abstract

**Background:**

Liver transplantation (LT) is the only treatment option for patients with advanced liver disease. Currently, liver donation to these patients, considering priorities, is based on the Model for End-Stage Liver Disease (MELD). MELD score is a tool for predicting the risk of mortality in patients with advanced liver disease. However, few studies have so far been conducted in Iran on the efficacy of MELD score of these patients.

**Objectives:**

This study reviews the present status of the MELD score and introduces a new model for optimal prediction of the risk of mortality in Iranian patients with advanced liver disease.

**Patients and Methods:**

Data required were collected from 305 patients with advanced liver disease who enrolled in a waiting list (WL) in Imam Khomeini Hospital from May 2008 to May 2009. All of the patients were followed up for at least 3 years until they died or underwent LT. Cox regression analysis was applied to select the factors affecting their mortality. Survival curves were plotted. Wilcoxson test and receiver operating characteristics curves for survival predictive model were used to compare the scores. All calculations were performed with the SPSS (version 13.0) and R softwares.

**Results:**

During the study, 71 (23.3%) patients died due to liver cirrhosis and 43 (14.1%) underwent LT. Viral Hepatitis (43.7%) is the most common cause of end-stage liver disease among Iranian patients. A new model (NMELD) was proposed with the use of the natural logarithms of two blood serum variables (total bilirubin and albumin) and the patients' age (year) by applying the Cox model:

NMELD = 10 × (0.736 × ln (bilirubin) – 1.312 × ln (albumin) + 0.025 × age + 1.776)

**Conclusions:**

The results of the Wilcoxon test showed that there is a significant difference between the usual MELD and our proposed NMELD scores (P < 0.001). Receiver operating characteristics curve for survival predictive model indicated that the NMELD score is more efficient compared with the MELD score in predicting the risk of mortality. Since serum creatinine was not significant in NMELD score, further studies to clarify this issue are suggested.

## 1. Background

Gastrointestinal and liver diseases are among the most common causes of morbidity in Iran and constitute a substantial proportion of mortality, which imposes enormous economic consequences (1, 2). Prognosis is an important part of the baseline assessment of any disease. It is not only the basis of the information that a physician provides to the patient, but is also the basis for any management method. Proper prioritization of patients waiting in queue for a liver transplant (LT) is needed and many methods have been developed for this purpose over several decades (3). In 1964, Child and Turcotte proposed a prognostic model for estimation of surgical risks in patients with advanced liver disease. Pugh et al. proposed a revision of this model in 1973. The modified Child-Pugh (CP) prognostic index has been widely used for risk stratifying of patients with cirrhosis and to assess the efficacy of beneficial procedures. At present, the CP classification is by far the most extensively applied system, as it is easy to use at the bedside (4-8). In 2000, the Department of Health and Human Services (DHHS) of the USA adopted the ‘Final Rule’. According to this rule, the primary guidelines for allocation of cadaver livers for transplantation should be based on medical urgency. Over the years, liver allocation policy has evolved from prioritizing liver transplant candidates based on their physical location (home, hospital or intensive care unit) to medical-based criteria (CP score) consistent with these guidelines (5, 9). The scoring system of the model for end-stage liver disease (MELD) has emerged as an excellent predictor of mortality on the waiting list (WL) (10-13). The combination of WL mortality risk and post-transplant mortality risk assessed by the MELD score and other factors can be used to estimate whether candidates are likely to derive a survival benefit from a LT. Recently, MELD score has replaced CP score in the USA for prioritizing donor liver allocation (14-27). The introduction of the MELD system for transplant allocation in the USA resulted in a 3.5% reduction in WL mortality while early survival of LT recipients remained unchanged despite the selection of more ill patients for transplantation (28). In 1991, the first LT was carried out in Iran. Currently, more than 100 LT are being carried out annually in several provinces including Tehran and Shiraz. At present, MELD and CP scores are widely used to stratify patients for LT in Iran (29-32).

## 2. Objectives

LT is recognized as the only way to treat patients with cirrhosis. The aim of this study was to determine the best time for LT and the factors influencing these patients’ mortality. Optimal allocation of the few available donor livers for WL is essential. In this study, the survival rate of patients awaiting LT for one, two and three years, and the related influential factors were examined. Here, we attempt to provide a more efficient scoring system than the previous for predicting the mortality risk of patients with advanced liver disease.

## 3. Patients and Methods

We evaluated all of the patients with advanced liver disease on the WL for LT in Imam Khomeini Hospital (Tehran, Iran) during May 2008 to May 2009. They were followed up for 3 years. The required data included demographic features, cause of cirrhosis and laboratory test results. Child-Pugh (CP) score was evaluated by five parameters: ascites, encephalopathy, bilirubin, prothomobin time and albumin. CP scores of all participants were calculated according to the method summarized in Table 1. CP scores ranged from 5 to 15 and allowed the categorization of the patients into 3 groups (23): a) CP < 7, b) 6 < CP < 9 and c) CP > 8.

**Table 1.** Child-Pugh Scores’ Calculating Method

	CP risk class points		
Criteria	A (1 point)	B (2 points)	C (3 points)
**Ascites**	None	Light	Large
**Serum bilirubin, mg/** **dL**	< 30	30-50	> 50
**Serum albumin, g/** **dL**	> 35	28-35	< 28
**Prothrombin** ** index**	> 54	44-54	< 44
**Encephalopathy**	None	Minimal	Advanced
**Total score**	5-6	7-9	10-15

MELD calculations were done on the natural logarithms (ln) of three variables in blood serum: total bilirubin, creatinine and the international normalized ratio (INR) of prothrombin. MELD score was calculated by the following formula:

MELD = 9.6 × ln (creatinin mg/dL) + 11.2 × ln (INR) + 3.8 × ln (bilirubin mg/dL) + 6.43

According to the United Network for Organ Sharing (UNOS) modifications, the laboratory values below 1.0 were rounded to 1 to avoid negative scores, and the maximum serum creatinine considered within the MELD equation was 4.0 mg/dL. In this paper, CP and MELD scores were calculated at the beginning of the study for all patients. Censorship group included the patients that underwent LT during this period or did not die by the end of the study. The univariate Cox regression models were applied separately to discriminate each significant factor. The adjusted survival and hazard functions were estimated using a multivariate Cox regression model. Factors associated with P-values of less than 0.20 for the previous stage (univariate Cox models) were candidates for multivariate analysis. In all models, factors with P Values < 0.05 were considered to be significant (33). The resulting predictive formula was normalized to the same scale as the MELD score by linear regression (34). To compare the new scores with the MELD scores, we used ROC curves for survival predictive accuracy. For a binary disease outcome, receiver operating characteristics curve (ROC) is a popular method for displaying sensitivity and specificity of a continuous diagnostic marker. However, some disease outcomes are time dependent, and ROC curves that vary as a function of time may be more appropriate. Standard Cox proportional hazard output can be used to obtain estimates of time dependent sensitivity and specificity, and time dependent ROC curves (35-37). In our study, these special Roc curves were used for predictive accuracy of survival models. About one-third of the patients (101), termed group 1, were randomly selected for score validation and the remaining patients (204) were named as Group 2. Group 2 was used for modeling of NMELD scores, and Group 1 was used for its validation.

## 4. Results

Of the 305 patients, 126 (41.3%) were females and 179 (58.7%) were males. The mean age of the patients was 40.67 (± 14.39) (Mean ± (Standard Deviation) (M ± SD) years. The age range was between 18 and 71 years. During the study, 71 (23.3%) patients died due to complications of liver cirrhosis and 43 (14.1%) underwent LT. Survival rate at one, two and three years after enrolling in the WL was 82%, 73% and 66%, respectively. Survival rate at one, two and three years for patients with MELD scores < 10 was 95%, 90% and 79%, for 9 < MELD < 20 was 89%, 79% and 75%, and for MELD > 19 was 55%, 40% and 27%, respectively.

**Figure 1.**
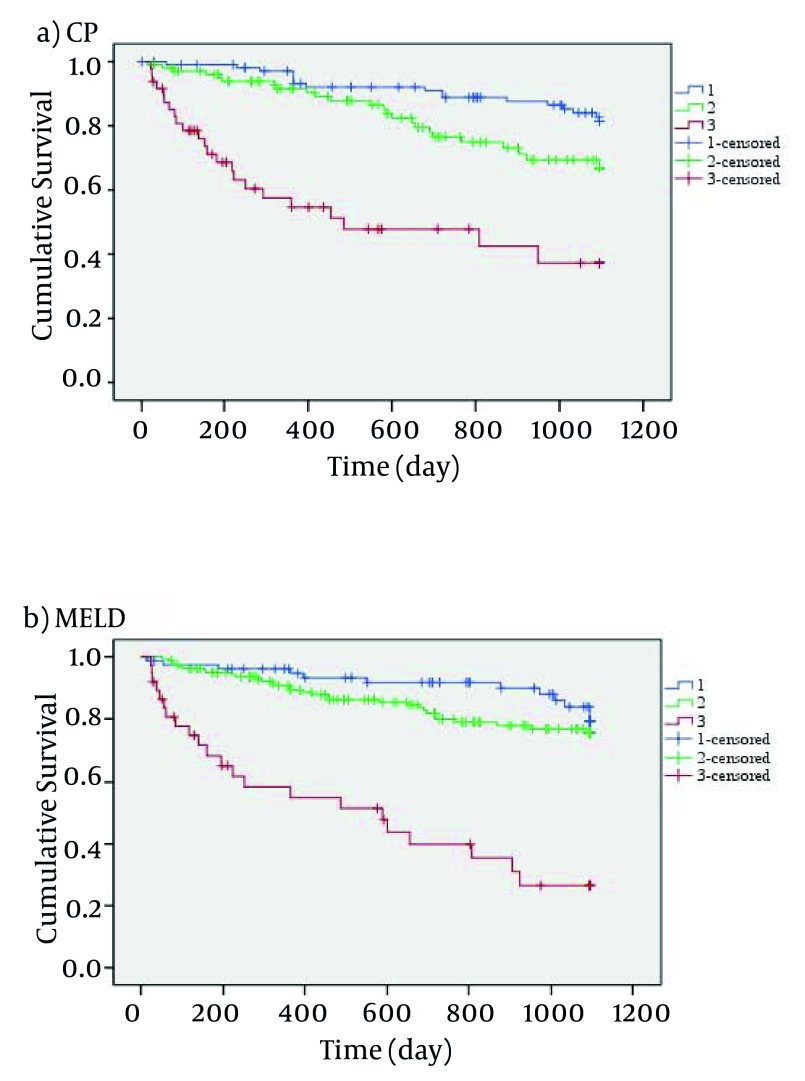
Kaplan–Meier Survival Curves for CP and MELD Scores a) Categories of CP scores: 1) CP < 6 as class A, 2) CP 7-9 as class B and 3) CP > 10 as class C b) Categories of MELD score: 1) MELD < 10, 2) MELD 10-19 and 3) MELD > 20

Figure 1 indicates that the mortality rate of patients with high MELD and CP scores was higher than those with lower MELD and CP scores. Using CP scores, 23.9% of patients were at stage A, 47.9% at stage B and 28.2% at stage C. Wilcoxon test showed no significant difference between MELD and CP scores (P = 0.112). In this study, the most frequent etiology of cirrhosis was hepatitis, and the rate of liver failure associated with viral hepatitis, autoimmune and cryptogenic was 34.7%, 16.4% and 22.6%, respectively. The detailed data of demographic variables are given in Table 2.

**Table 2.** Demographic, Clinical, Biochemical Features of Patients Awaiting Liver Transplantation

Demographic	Total	Group 1	Group 2
**Gender, No. (%)**			
Male	180 (58.7)	59 (58.4)	121 (59.3)
Female	125 (41.3)	42 (41.6)	83 (40.7)
**Education, No. (%)**			
Primary	94 (30.8)	36 (35.7)	58 (28.4)
Secondary	168 (55.1)	52 (51.5)	116 (56.9)
High education	43 (14.1)	13 (12.8)	30 (14.7)
**Marriage status, No. (%)**			
Single	83 (27.2)	24 (23.8)	59 (28.9)
Married	216 (70.8)	75 (74.2)	141 (69.1)
Divorced or widow	6 (2.0)	2 (2.0)	4 (2.0)
**Family history of the liver disease, No. (%)**			
Yes	36 (11.8)	17 (16.8)	19 (9.3)
No	269 (88.2)	84 (83.2)	185 (90.7)
**Cause of cirrhosis, No. (%)**			
Hepatitis B virus	66 (21.6)	27 (26.7)	39 (19.1)
Hepatitis C virus	40 (13.1)	14 (13.9)	26 (12.7)
Auto Immune	50 (16.4)	19 (18.8)	31 (15.2)
Cryptogenic	69 (22.6)	17 (16.8)	52 (25.5)
Alcohol	2 (0.7)	1 (1.0)	1 (0.5)
PSC	27 (8.9)	5 (5.0)	22 (10.8)
PBC	12 (3.9)	3 (3.0)	9 (4.4)
Others	39 (12.8)	15 (14.8)	24 (11.8)
**Clinical Ascites, No. (%)**			
None	201	69 (68.3)	132 (64.7)
Light	93	26 (25.8)	67 (32.8)
Large	11	6 (5.9)	5 (2.5)
**Encephalopathy, No. (%)**			
None	137 (44.9)	58 (57.4)	81 (39.7)
Minimal	96 (31.5)	38 (37.6)	58 (28.4)
Advanced	72 (23.6)	5 (5.0)	65 (31.9)
**Biochemical, mean ± SD**			
Serum bilirubin	4.290 ± 6.394	4.200 ± .480	4.466 ± .587
Serum creatinine	0.990 ± 1.177	1.024 ± .101	0.924 ± .056
Serum albumin	3.686 ± 2.285	3.617 ± .123	3.821 ± .318
**INR for prothrombin time**	1.695 ± .744	1.661 ± .054	1.761 ± .074

In Group 2, 83 (40.7%) were females and 121 (59.3%) were males. 48 (23.5%) patients in this group died due to complications of liver cirrhosis and 27 (13.2%) underwent LT. The mean age of the patients was 41.6 (± 0.98) (Mean ± (Standard Error) (M ± (SE)) years. The age range was between 18 and 65 years. In univariate analysis, continuous variables showed a significant association with the patients’ age (P = 0.009), ln (bilirubin) (P < 0.001), ln (albumin) (P < 0.001) and ln (INR) (P < 0.001). Categorical variables revealed an association between ascites (P < 0.045) and encephapolathy (P = 0.013). The results of univariate analysis are shown in Table 3.

**Table 3.** Univariate Analysis of Risk Factors Associated With Mortality in Cirrhotic Patients

Variables	Regression coefficient	Regression coefficient standard error	P value
**Ascites (+)**	0.496	0.242	0.045
**Encephalopathy (+)**	1.445	0.582	0.013
**Age**	0.014	0.006	0.009
**ln** **(bilirubin)**	0.818	0.131	< 0.001
**ln(albomin)**	-1.836	0.469	< 0.001
**ln** **(INR)**	1.555	0.341	< 0.001

Cox multiple regression analysis indicated that there is a statistically significant correlation between ln (bilirubin), ln (albumin) and age, and the risk of mortality. The results of multivariate analysis are shown in Table 4.

**Table 4.** Multivariate Analysis of Risk Factors Associated With Mortality in Cirrhotic Patients

Variables	Regression coefficient	Regression coefficient standard error	P- value
**ln(bilirubin)**	0.979	0.153	< 0.001
**ln(albomin)**	-1.745	0.469	< 0.001
**Age**	0.033	0.011	0.010

The score of the optimal model was calculated via the below formula:

A = 0.979 × ln (bilirubin) – 1.745 × ln (albumin) + 0.033 × age

By linear regression, we found that the best linear fit between A (as independent variable) and the MELD score (as dependent variable) was provided by a line with the slope of 7.519 and an intercept of 17.661. The correlation between the two was good with R = 0.560. Based on the above analysis, the scores obtained from the model can be derived from the below formula. Here, we’ve termed it the ‘New MELD (NMELD)’ score:

NMELD = 10 × (0.736 × ln (bilirubin) – 1.312 × ln (albumin) + 0.025 × age + 1.776)

To avoid negative scores, serum bilirubin values below 1.0 were rounded to 1 and serum albumin values over 5.4 were rounded to 5.4; the obtained scores were rounded to the nearest integer. All analyses for modeling of NMELD were done based on the data collected from Group 2. In Group 1, 42 (41.6%) individuals were females and 59 (58.4%) were males. 23 (22.8%) patients in this group died due to complications of liver cirrhosis and 16 (15.8%) underwent LT. The mean age of the patients was 40.2 (± 1.53) years. The age range was between 18 and 71 years.

**Figure 2.**
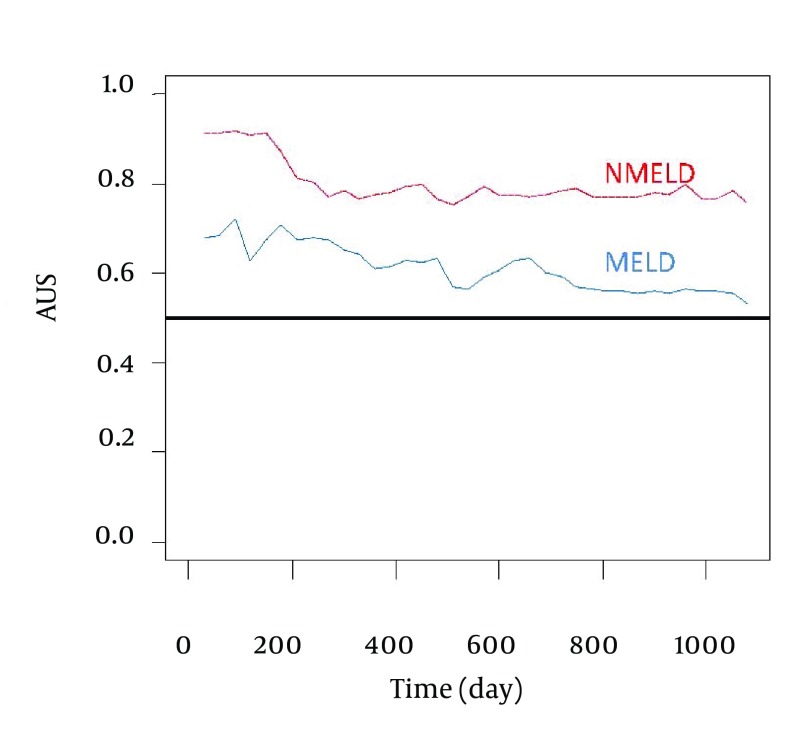
Accuracy of the NMELD Score (Dash Line) Using the Covariates of ln (Bilirubin), ln (Albumin) and age vs. MELD (Solid Line) Score Using the Covariates of ln (Bilirubin), ln (INR) and ln (Creatinine). Lines Plot the Estimates of Incident/Dynamic AUC (t) Versus Time Under the Assumption of Proportional Hazards.

**Figure 3.**
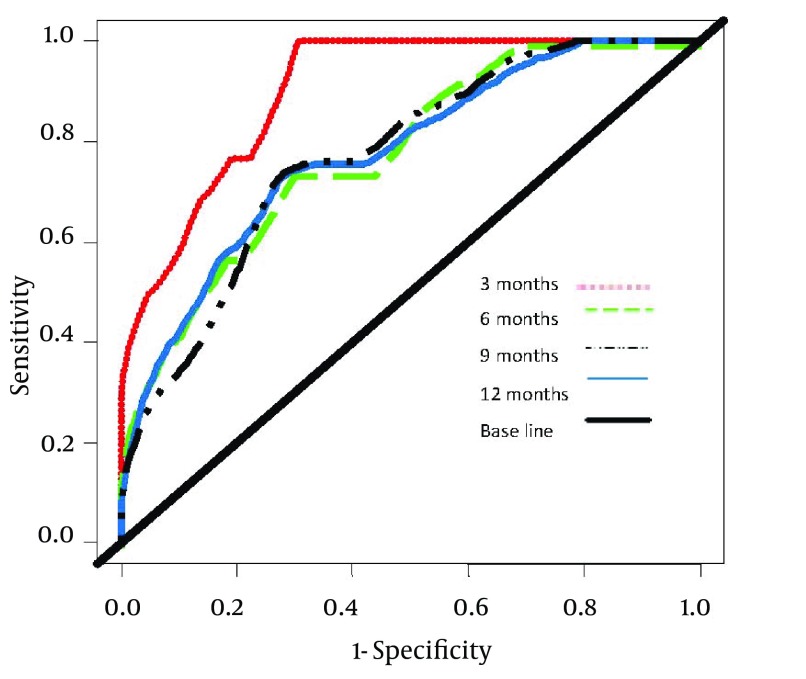
Comparison of the Area Under ROC Curves (AUC) for Predicting the Risk of Mortality at 3 (AUC = 0.916), 6 (AUC = 0.811), 9 (AUC = 0.788) and 12 (AUC = 0.780) Months for NMELD Scores.

Comparison of accuracy of the NMELD Score (Dash Line) Using the Covariates of ln (Bilirubin), ln (Albumin) and age vs. MELD Score (Solid Line) Using the Covariates of ln (Bilirubin), ln (INR) and ln (Creatinine). Lines Plot the Estimates of Incident/Dynamic AUC (t) Versus Time Under the Assumption of Proportional Hazards. Figure 2 shows that the NMELD score is superior in predicting the risk of mortality. Since the AUC values for MELD score are about 0.5, this model is inefficient for predicting the risk of mortality. Figure 3 shows that NMELD score is more accurate than MELD score in assessing the risk of mortality in the short term. Comparisons of the NMELD and MELD scores were performed based on data collected from Group 1.

## 5. Discussion

LT has been accepted as an effective method for the recovery of health in patients with advanced liver disease. However, the increasing discrepancy between the number of patients on WLs and the number of available donor livers has a major impact on the mortality of these patients (38). Disease severity at the time of listing has been demonstrated as an important predictor of WL death. Prognostic assessment of patients with liver cirrhosis is a vital subject that often challenges the clinicians. CP score is by far the most extensively used both in clinical practice and clinical research, and has stood the test of time for nearly 40 years. Recently, the MELD score has replaced the CP score in the United States for prioritizing liver donor allocation. Como et al. (39) showed that the use of the MELD score produced an advantage for Hepatocellular Carcinoma (HCC), but about one in every 11 viral hepatitis patients may be harmed using this scoring system.

**Figure 4.**
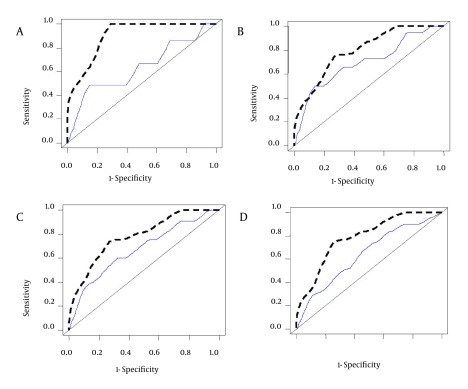
Comparison of the Area Under the ROC Curves (AUC) for Predicting the Risk of Mortality at 3 (A), 6 (B), 9 (C) and 12 (D) Months Between NMELD (Dash Line) and MELD (Solid Line). AUC for Prediction of the Risk of Mortality at 3 (AUC = 0.916), 6 (AUC = 0.811), 9 (AUC = 0.788) and 12 (AUC = 0.780) Months for the NMELD Score and AUC for Prediction of the Risk of Mortality at 3 (AUC = 0.636), 6 (AUC = 0.697), 9 (AUC = 0.672) and 12 (AUC = 0.640) Months for MELD Score.

Among the 305 patients in this study, who were listed for LT from May 2009 to May 2012, the most common etiology of liver disease was viral hepatitis (34.7%). This is in contrast with the findings of the Malinchoc et al. study, conducted at a medical center in the USA where alcoholic liver cirrhosis was the most prevalent (38.8% to 90.5%) followed by viral hepatitis (4.8% to 10.4%), and also with the results of a study by Sumskiene et al. in Lithuania where alcoholic liver cirrhosis was the most prevalent (28.9%) followed by viral hepatitis (17.8%) (40, 41). This major difference is mainly due to the prohibition of alcohol by Islamic rules; therefore, alcoholic liver cirrhosis is not common in Iran. According to the data shown in Figure 4, NMELD score is superior to the MELD score in accurately predicting the risk of mortality in Iranian patients with advanced liver disease.
